# Promote One, Inhibit the Other: A Single Pathway Controls Axon and Dendrite Growth, Oppositely

**DOI:** 10.1371/journal.pbio.1001575

**Published:** 2013-06-04

**Authors:** Richard Robinson

**Affiliations:** Freelance Science Writer, Sherborn, Massachusetts, United States of America

To make a functioning neuron, you need both a dendrite to receive signals and an axon to send them. Therefore a shared pathway, which stimulates the growth of both, might come in handy. But growing one or the other alone could also be beneficial, and so it would make sense to have dedicated pathways that could stimulate either process independently. Indeed, such shared and dedicated neuronal growth pathways have both been identified. But then there are times when it might be helpful to promote growth of one while inhibiting the other. In a new study in *PLOS Biology*, Xin Wang, Bing Ye, and colleagues demonstrate the existence of this kind of bimodal control in flies, and identify the molecular pathway that makes it possible.

The authors focused on a signaling pathway known to control axon terminal growth. In flies, two key members of the pathway are Highwire and Wallenda. These two interact, in that Highwire suppresses expression of Wallenda, and loss of Highwire causes axonal overgrowth that can be reversed by loss of Wallenda.

When the authors inactivated Highwire in a set of larval body wall neurons, not only did they observe the expected axon terminal overgrowth, but they also saw dramatic reduction of dendritic growth, leading to dendrites that were both shorter and less highly branched than normal. The neurons were otherwise normal, displaying expected molecular markers and finding their way to their appropriate targets on the fly nerve cord. Inactivating the gene for Wallenda reversed the axonal outgrowth, as expected, and also the inhibition of dendrite growth. Conversely, excess of Wallenda favored axon terminal growth and inhibited dendrite growth, strongly suggesting that Highwire and Wallenda work within a single pathway to regulate the growth of both axon and dendrite, with Highwire promoting dendrites and Wallenda axons.

At some point, however, the pathway must split, in order to produce its divergent effects. Other experiments have shown that the Fos protein is a downstream effector of Wallenda. Here, inactivation of *fos* prevented axon terminal overgrowth when Wallenda was overexpressed, indicating it plays a key role in the axonal branch of the pathway. But Fos had no effect on dendritic growth, so the authors speculated that perhaps that branch involved one or more transcription factors with roles in growth of dendrites. They found that levels of one, called Knot, were reduced by either inactivation of Highwire or overexpression of Wallenda. Restoring Knot reversed the dendritic, but not the axonal, defects caused by Highwire inactivation.

In sum, then, it appears that when Highwire suppresses expression of Wallenda, expression of Knot increases and leads to the elaboration of the dendritic tree. Conversely, when Wallenda expression is elevated, *fos* is activated and promotes axon terminal outgrowth, while Knot expression is reduced, inhibiting growth of dendrites. One possible benefit of this bimodal control system is to coordinate the response to axonal injury. Axon regrowth requires cellular resources of membrane and cytoskeletal proteins, which are abundant in the dendrite. It may be that the Highwire/Wallenda system maximizes the ability of the neuron to move resources to the axon by suppressing their use in the dendrite. In support of this possibility, shrinkage of dendrites is observed following experimental induction of axon damage. A better understanding of how neurons balance the competing needs of their two critical components should lead not only to a better understanding of normal neuronal growth and development, but also to better strategies for aiding neuronal repair after traumatic injury.

**Wang X, Kim JH, Bazzi M, Robinson S, Collins CA, et al. (2013) Bimodal Control of Dendritic and Axonal Growth by the Dual Leucine Zipper Kinase Pathway. doi:10.1371/journal.pbio.1001572**

**Figure:**
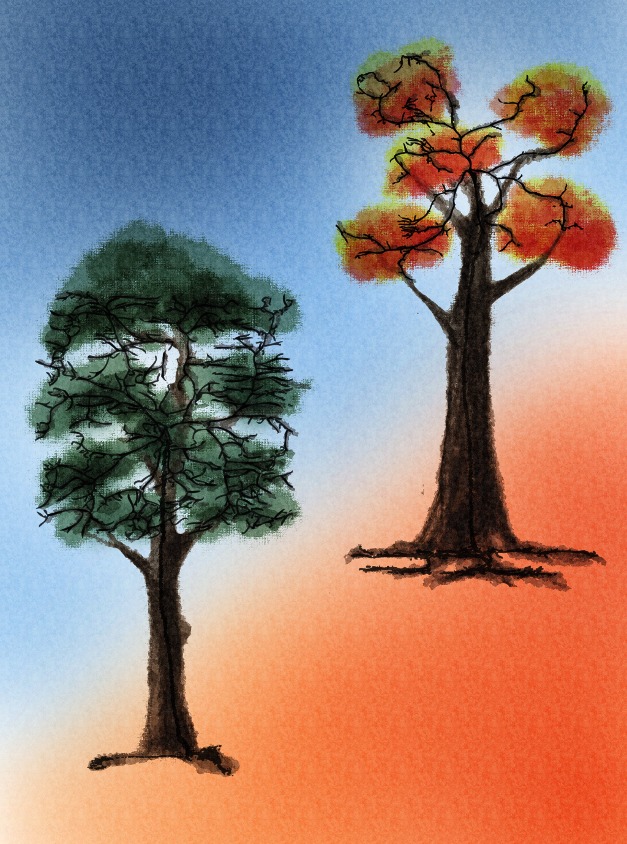
A functioning neuron (symbolized here as a tree) has both dendritic arbors to receive signals and axonal branches to send them. **The activity of a single molecular pathway (shown as the orange slope) determines whether a neuron grows more dendrites and fewer axons (left tree) or more axons and fewer dendrites (right tree).** Image credit: Xin Wang.

